# COVID‐19 transmission flow through the stigmatization process in Bangladesh: A qualitative study

**DOI:** 10.1002/lim2.52

**Published:** 2021-12-04

**Authors:** Md. Shahgahan Miah, Md. Razib Mamun, S. M. Murshid Hasan, Md. Golam Faruk Sarker, Muhammad Salim Miah, Md. Gias Uddin Khan, Ashraful Kabir, Mohammad Ainul Haque, N. M. Rabiul Awal Chowdhury

**Affiliations:** ^1^ Department of Anthropology Shahjalal University of Science and Technology Sylhet Bangladesh; ^2^ Department of Public Health and Health Systems Nagoya University Graduate School of Medicine Nagoya Japan; ^3^ Department of Society and Health Faculty of Social Sciences and Humanities Mahidol University Salaya Thailand; ^4^ Department of Anthropology University of Rajshahi Rajshahi Bangladesh; ^5^ Department of Economics Shahjalal University of Science and Technology Sylhet Bangladesh; ^6^ School of Public Health and Preventive Medicine Monash University Melbourne Australia; ^7^ Department of Anthropology Comilla University Comilla Bangladesh

**Keywords:** Bangladesh, Coronavirus, Coronavirus disease, Stigma, Transmission

## Abstract

**Introduction:**

Coronavirus disease (COVID‐19) patients and survivors face stigma, discrimination, and negligence. The motives for and the different types and consequences of COVID‐19‐related stigmatization remain underexplored in Bangladesh. Therefore, this study examined how the COVID‐19 stigmatization process is interlinked with transmission flow.

**Methods:**

Using a qualitative research design, we conducted 20 in‐depth interviews with infected and suspected caregivers and five key informant interviews with physicians, local media representatives, leaders, law enforcement officials, and local administrative officials in three divisional cities of Bangladesh. We performed thematic analysis to analyze the data.

**Results:**

Participants expressed their experiences with multiple subthemes within three themes (stigma related to symptoms, stigma associated with isolation and quarantine, and stigma associated with health services). Participants reportedly faced stigma, for example, exclusion, hesitation to interact, avoidance, bullying, threat, and negligence caused by misinformation, rumors, and fear. Stigmatized individuals reportedly hid their symptoms and refrained from seeking healthcare services, contributing to COVID‐19 transmission flow.

**Conclusion:**

Revealed insights may contribute to effective prevention, control, and management of such an emerging pandemic. Further in‐depth exploration of such stigmatization process will enrich unexpected outbreaks management effectively.

## 1 INTRODUCTION

The coronavirus disease (COVID‐19) poses unprecedented threats to global public health. Until August 2021, COVID‐19 caused 4.5 million deaths and negatively affected everyday life worldwide.^1^ In specific, the COVID‐19 has disrupted daily life activities, socioeconomic systems, and access to healthcare in low‐, middle‐, and high‐income countries.^2^, ^3^ Individuals possess limited knowledge about this novel virus, resulting in fear, discrimination, and stigma. During a pandemic or an epidemic, fear exacerbates stress and psychological symptoms.^4^, ^5^ Because, the novelty and rapid spread of the COVID‐19 and the associated uncertainty, fear levels have been higher during the ongoing pandemic than in past epidemics.^6^ During a pandemic, individuals are vulnerable to social isolation, a lack of access to healthcare services, harassment, and bullying. In addition, individuals with active COVID‐19 symptoms have been found to hide their symptoms when accessing healthcare services because of their fear of stigmatization.^7^ Understanding the COVID‐19 transmission flow and the associated stigma process is crucial to respond to this pandemic effectively. To date, little has been explored about the COVID‐19 transmission flow interlinking with the stigma process.

It is essential to understand the definition of stigma to identify the COVID‐19 transmission flow through the stigma and its internalization process. The idea of stigma owes a great deal to Goffman, who viewed stigma as a social construction of identity, the situation of the individual who is disqualified from full social acceptance.^8^ Stigma is located within the stigmatized person and the social context that explains a devaluing attribute. Goffman further identified that the stigma occurs as a discrepancy between the “virtual social identity” where individuals are characterized by society and “actual social identity” where a person possessed the attributes.^8^ A recent study identified that the COVID‐19 patients are being treated as ‘other’ that can also be categorized them as stigmatized.^9^ The power relation is central to construct the stigmatization where the ordinary people (such as a healthy individual) control the social, economic, and political power.^10^ Therefore, stigmatization is attributed to society's dominant group, and the stigmatizing attributes are broadly identified in the culture. The COVID‐19 related stigmatization pattern could be different based on the sociocultural context. Commonly sociocultural context represents those forces and reality which influence and often control human thoughts and practices. Study participants' perception, knowledge, belief, religious practices, educational level, political and economic position, and respective attitudes and behaviors were mostly demonstrated in this study in sociocultural context.

The COVID‐19‐related fear and stigma threaten the lives of healthcare providers, patients (suspected and infected), their family members, and survivors.^11^, ^12^ Past outbreaks of infectious diseases are associated with discrimination and stigma across different populations.^12^ Notably, social media panic has spread faster than the virus and directly contributes to fear and stigma.^13^, ^14^, ^15^ In a recent study on the COVID‐19‐related infodemic on online platforms, the researchers identified 2311 reports of rumors and stigma from 87 countries.^16^ During a pandemic, stigma exerts several adverse effects (e.g., exacerbate physical, social, and psychological distress) on infected individuals. The stigmatization of healthcare providers may adversely affect healthcare provision and efforts to control the spread of the virus.^11^, ^17^

Experience gained during past epidemics (e.g., human immunodeficiency virus/acquired immunodeficiency syndrome, HIV/AIDS) and outbreaks (e.g., Ebola and other transmissible diseases) suggest that pandemic responses often focus on biomedical issues and overlook the relevant sociocultural, economic, and political ramifications.^18^ A limited understanding of the impact of such diseases poses challenges to effective policymaking and implementation. The sociocultural dynamics associated with the COVID‐19 pandemic have received little attention.^18^, ^19^ There is a lack of evidence in this regard, especially in the context of low‐ and middle‐income countries. Researchers have emphasized that cultural factors should be prioritized to understand the nature of stigma because stigma experiences are local and often influenced by sociocultural factors.^20^, ^21^

By August 2021, more than 1.5 million confirmed cases and around 25,000 deaths had been reported in Bangladesh.^1^ Further, incidents of verbal abuse and stigmatization, including reticence to treat non‐COVID‐19 patients, abandonment by family, forced eviction from home, denial of burial, and harassment, have been observed.^22^ The types and consequences of COVID‐19‐related stigmatization witnessed in Bangladesh may differ from those reported in other countries. Therefore, there is an urgent need to examine the different kinds of COVID‐19‐related stigmatization in Bangladesh to create awareness among the general public, develop more effective infection control strategies, and ensure equitable healthcare. Thus, this study aims to explore the COVID‐19‐related transmission flow and its connections with stigmatization among a heterogeneous group of individuals in Bangladesh.

## 2 METHODS AND MATERIALS

### 2.1 Study design and settings

This study adopted the qualitative study design to explore the process and consequences of stigmatization among a heterogeneous group. Because the qualitative research reveals the complex phenomenon in the healthcare settings faced by the recipients, healthcare providers, policymakers, and even the clinicians.^23^ Data were collected from three divisional cities (largest administrative zone), namely Dhaka, Sylhet, and Chattogram in Bangladesh. Notably, a higher COVID‐19 infection and death rate was reported in these three cities. The study was conducted from April to October 2020.

### 2.2 Study population and sampling strategy

We recruited our study participants from the three divisions of Bangladesh to capture their heterogeneity in socioeconomic status (Table 1). A purposive sampling strategy was used to ensure the participants’ heterogeneous backgrounds, including age, gender, education, health status (COVID‐19 infected and suspected), and occupation. We included study participants aged 18 years or older and able to speak voluntarily. We used the interpersonal communication skills of the study team to identify the potential participants.

**TABLE 1:** Data collection tools, field sites, and study participants (*n* = 25)

Field sites	Data collection tools	Participants
Dhaka	IDI	Infected patient (*n* = 3) Suspected patient (*n* = 3) Family caregiver (*n* = 3)
	KII	Law enforcement official (*n* = 1) Administrative official (*n* = 1)
Sylhet	IDI	Infected patient (*n* = 2) Suspected patient (*n* = 2) Family caregiver (*n* = 2)
	KII	Hospital physician (*n* = 1)
Chattogram	IDI	Infected patient (*n* = 2) Suspected patient (*n* = 1) Family caregiver (*n* = 2)
	KII	Frontline local leader (*n* = 1) Media representative (*n* = 1)

*Note*. IDI = in‐depth interview, KII = key informant interview.

### 2.3 Data collection tools and procedures

We conducted 20 in‐depth interviews (IDIs) with the suspected or confirmed COVID‐19 patients and their family caregivers who experienced stigma by their family members, neighbors, and other associates. Moreover, we conducted five key informant interviews (KIIs) with physicians, local media representatives, leaders, law enforcement officials, and local administrative officials. Instead of focusing on one or two professional groups, we sought to achieve professional heterogeneity when selecting these key informants.

A research team experienced in qualitative methods and techniques collected the data. We developed semistructured interview guidelines for the IDIs and KIIs. The guidelines entailed questions about the events, processes, and experiences associated with stigmatization. All the study participants voluntarily participated in this study. Both telephone‐based and face‐to‐face interviews were conducted following participants' choice to ensure interviewee and interviewer health safety and compliance with travel restrictions. In addition, we conducted 12 telephone interviews as the participants avoided face‐to‐face talking. The interviews were conducted in Bangla (the mother tongue of the interviewers and interviewees). On average, each interview lasted for 45–60 min. Several follow‐up telephone calls were made to collect missing data and further explore specific issues with some respondents. We adopted the data saturation principle (i.e., the point beyond which no new information, theme, or dimension emerged) to determine the required sample size.^24^, ^25^

### 2.4 Data analysis

We conducted a thematic analysis to analyze the data.^26^, ^27^ We followed the inductive approach for thematic analysis. First, the audio‐recorded interviews were transcribed verbatim and translated into English. Then, three researchers repeatedly read the transcripts independently to familiarize themselves with primary data and its meaning. Therefore, a primary open and axial code list was generated. Subsequently, we created clusters based on these codes. Finally, three researchers created themes and subthemes based on the emergent clusters. We randomly reviewed a few interview transcripts to identify and rectify errors in the axial codes. We used memo writing tools to map our data analysis and extract the meaning from primary data.^28^ Therefore, the meaning of data helped identify the contributors and process of stigmatization for framing our conceptual framework. Disagreements were resolved through discussions among the team members. The entire data analysis procedure was undertaken manually.

### 2.5 Ethical considerations

This study followed the ethical agreement of the Helsinki declaration. This research obtained ethics approval by the Research Ethics committee of the Anthropology department of Shahjalal University of Science and Technology. We obtained verbal consent over the phone for mobile interviews about voluntary participation before starting the interview. We used the pseudonyms of the participants to ensure the privacy and confidentiality of participants.

## 3 RESULTS

**FIGURE 1:**
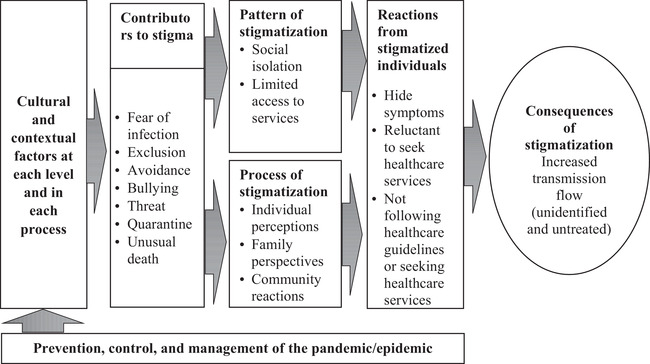
Conceptual framework of the stigmatization of coronavirus disease patients to transmission flow within the community

Figure 1 presents the conceptual framework that was developed based on the present findings. Through various interview sessions with the study participants, bullet points were evolved during the conversation. A list of contributors, patterns, and processes of stigmatization and respective reactions was compiled from various interview sessions. Memo‐writing methods were adopted here in this listing, and an inductive approach was followed in the compilation. Figure 1 illustrates how stigmatization stimulates COVID‐19 transmission flow. Cultural and socioeconomic factors are integral aspects of epidemic control protocols (in addition to clinical factors). In Bangladesh, COVID‐19‐related information, which included frightening messages and perpetuated misinformation, was widely circulated. Data from various sources puzzled general people. Because of sociocultural factors, most individuals lack the ability to judge the authenticity of incoming information. Consequently, they disseminated misinformation to others. This contributed to stigmatization, fueled by fear of infection, exclusion, avoidance, bullying behavior, threats, quarantine, and unusual death. Stigmatization manifested in different forms (e.g., social isolation, economic vulnerability, and limited access to services) and was supported by individual perceptions, interpretations, family perspectives, and community reactions, which again were shaped by cultural, contextual, and societal factors dynamics. In response to stigma, individuals may hide their symptoms, refrain from seeking healthcare services, and pay no attention to healthcare guidelines. However, such behaviors exacerbated the crisis by increasing transmission flow.

### 3.1 Stigma related to symptoms

Commonly reported COVID‐19 symptoms included severe diarrhea, high fever, sleeplessness, throat dryness, sneezing, pain, trigger fingers, a loss of taste and smell, and breathing difficulties. These symptoms created distress in the individuals with suspected or confirmed COVID‐19 and their family members. In addition, these symptoms brought profanity to the suspected or infected individuals and the family members. All the participants reported that suspected and infected individuals hid their symptoms from their household and family members, individuals in their workplace, and even doctors during their visits to hospitals and clinics. These behaviors were motivated by fear of being ostracized by family members, neighbors, and healthcare providers.

#### 3.1.1 Fear of being infected by other patients

Fear of being infected by other infected patients emerged as a factor that prevented individuals from visiting a diagnostic center. Further, the participants reported that individuals waiting in queues did not practice social distancing or wear masks. Moreover, the informants noted that many visitors were coughing but not wearing masks. Therefore, many individuals reportedly avoided visiting hospitals and diagnostic centers to avoid contact with infected and suspected individuals. For example, one IDI participant made the following statement:

Visitors wear a mask when they talk to others and move from one place to another. But they put their mask in their pocket when they sit on a chair. (Male, 38 years, suspected patient, Chattogram)

#### 3.1.2 Tendency to not disclose symptoms

Symptomatic informants reported that they were living with the fear of death. The media, especially television channels, highlighted the high rate of COVID‐19 infections and deaths. Additionally, some participants reported that distressing messages and video footage depicting the harassment of COVID‐19 patients had flooded their social media pages (e.g., Facebook, Twitter). Such news reports exacerbated fear among the general public and motivated them to hide any COVID‐19 symptom.

I felt that I was going to die and (I was) so depressed hearing the news about Italy's death rates on the television. (Male, 41 years, suspected patient, Dhaka)

In addition, some participants reported that news reports of person‐to‐person transmission had exacerbated fear among family members. This in turn had resulted in avoidance behaviors and the neglect of symptomatic individuals with suspected COVID‐19. However, the behaviors of family members during the pandemic also contributed to the nondisclosure of COVID‐19 symptoms.

I have had breathing trouble since my childhood, and it was normal to my family members. However, the COVID‐19 news from the media made my spouse fearful of being close to me. Now, I am staying alone in a separate room. (Male, 40 years, suspected patient, Dhaka)

Key informants also reported that many infected patients hid their symptoms when they visited hospitals. Potential reasons include noncooperation and a lack of access to the healthcare services provided by the respective facility. These risky behaviors were demoralizing to service providers and adversely affected their motivation to provide proper care. Moreover, many doctors, nurses, and other healthcare providers contracted the disease through contact with nondisclosing patients.

Some patients were found to be COVID‐19‐positive after diagnosis, but they did not disclose their symptoms. They came and spoke like other general patients. But diagnosis and consultation revealed high fever with breathing difficulties. (Male, 45 years, hospital physician, Sylhet)

### 3.2 Stigma related to isolation and quarantine

Isolation and quarantine were unfamiliar terms to two‐third of the IDI participants. A large majority of the participants did not know about the terms “isolation and quarantine” in their lifetime. These terms were widely used in all COVID‐19 related announcements and news. No similar terms in the native language were used. The meaning of these terms was expressed differently by the participants. Such terms resulted in more fear and negligence.

#### 3.2.1 Noncooperation from neighbors and local leaders

Families with COVID‐19‐positive patients faced unpleasant behaviors from their neighbors. Sometimes, community members compelled families with a patient to quarantine themselves. Moreover, family members were not allowed to leave their homes even during an emergency. Neighbors often prevented patients from getting tested or seeking treatment. Because of such a state of affairs, one could neither seek treatment nor receive support from those around them. One KII participant described this situation as follows:

Having positive patients is like a great sin, the punishment for which is sitting at home and dying. (Male, 55 years, Frontline local leader, Chattogram)

The local leaders had placed a red flag outside the houses of infected individuals. Then, using a loudspeaker, they announced that the respective individuals were under quarantine so that others could identify and maintain a distance from them. One IDI participant provided the following description:

They (neighbors) accused me of bringing deadly things (coronavirus) to my village. Nobody shows my family members and me any mercy for this. (Male, 36 years, suspected patient, Dhaka)

Another participant provided the following account in IDI:

Neighbors asked us to stay at home, and they announced through a loudspeaker so that nobody could visit us. That makes us mentally weak. (Female, 40 years, Caregiver, Dhaka)

Such activities had created fear among the people and motivated them to hide their symptoms and not practice social distancing while interacting with others. As a result, the participants reported that suspected individuals tended to hide their symptoms to keep themselves safe, avoid social exclusion, and avoid being subjected to the restrictions imposed on infected individuals.

### 3.3 Stigma related to health services

Access to diagnostic services, healthcare facilities, and treatment emerged as an influential factor in the stigmatization of suspected and infected patients. Stigmatized people had limited access to healthcare facilities for getting not only the COVID‐19 related services but also other general and emergency health services. The healthcare facility centers and the physicians stopped providing healthcare services to the suspected and infected individuals because of the fear of infection of the coronavirus. Therefore, the other general and emergency patients also cannot access the healthcare service facility center. Because of this complex interplay between stigma and healthcare access, patients were forced to adopt healthcare practices such as adopting indigenous home remedies, self‐medicating, and visiting untrained healers, homeopaths, and, sometimes, expensive private clinics or hospitals.

#### 3.3.1 Lack of access to healthcare facilities

A lack of access to diagnostic and healthcare services emerged as an influential factor in the stigmatization process. The media had reported that COVID‐19 tests had not been adequately validated and a shortage of test kits and protective equipment. These factors deterred symptomatic patients from visiting service providers and influenced symptom disclosure. For example, one IDI participant made the following statement:

During the first few days, I experienced mild fever, a runny nose, and breathing difficulties. After three days, the situation turned into a nightmare as we could not get tested failed to manage the test. We visited most private, and public hospitals/clinics for a diagnosis but could not get tested. (Male, 38 years, suspected patient, Dhaka)

The participants reported that HIV‐positive patients with COVID‐19 symptoms shouldered a double burden of victimization. Health service providers did not cooperate to access health facilities. One IDI participant provided the following description:

You have a virus (HIV). How could you expect to get tested for COVID‐19 here? You will not be tested here.” They did not even listen to my words. (Male, 35 years, suspected patient, Chattogram)

#### 3.3.2 Restrictions

Hospitals designated for the treatment of COVID‐19 patients had imposed strict restrictions on patient movement and visitor access. Therefore, suspected patients with mild symptoms were reluctant to seek healthcare services. The participants compared such hospitals to prisons. Nevertheless, most participants continued to engage in their usual activities until their physical health condition deteriorated.

## 4 DISCUSSION

This study focused on two major aspects of stigmatization: (a) processes underlying stigmatization and (b) the role of stigma in disease transmission and healthcare access. A wide range of factors were associated with the experience of stigmatization during the COVID‐19 pandemic in Bangladesh. The following factors contributed to stigma: fear of infection, social exclusion, neglect by professional and family caregivers, and unusual death. Threats from local leaders, neighbor bullying behaviors, and harsh treatment from different sources had resulted in distress. Past studies have focused on stigma rooted in societal factors.^7^ Still, this study has identified new forms of stigma perpetuated by healthcare providers too (e.g., a lack of access, carelessness, negligence, and failure to provide treatment). Because of economic insecurity and low educational level, the participants were concerned about isolation, movement restriction, and quarantine protocols. Access to test, long wait for diagnosis, better health services, and other insecurity of treatment added to the stigma. Fear and poor awareness contribute to stigma, which has significant psychological effects during emergencies such as epidemics and pandemics.^4^, ^5^, ^6^, ^7^

Understanding the stigmatization process is very important for the prevention, control, and management of infectious diseases. Consistent with past observations, we found that disease outbreaks are closely associated with stigma, which accelerates infection spread and poses threats to life.^11^, ^12^ Exploring the sociocultural and contextual factors involved in the stigmatization process yields a comprehensive portrait of such phenomena. As documented in the literature, such findings have significant implications for the management of disease outbreaks.^18^, ^19^ If cultural factors are not adequately considered and assessed, awareness and behavior change interventions are at high risk for failure. The present findings have significant implications for policymaking regarding COVID‐19 management in Bangladesh because deciphering the role of sociocultural factors enriches our understanding of stigma experiences.^20^, ^21^

Stigmatization is a distressing experience that can lead to changes in traditional cultural practices. Therefore, it is important to understand the cultural factors involved in the stigmatization process. Without addressing the pertinent sociocultural factors, the prevention, control, and management of public health emergencies (e.g., epidemic and pandemic) will prove to be very difficult. Because of the strong influence of mass media and popular culture, individuals may fail to engage in hygiene behaviors that protect against COVID‐19 transmission. The present findings suggest that misinformation and an inability to judge the authenticity of COVID‐19‐related information contribute to stigma. Improper public health communication and the absence of a comprehensive plan of action are underlying reasons. Instead of blaming the general public for their ignorance, mass awareness campaigns should be conducted to address these issues. Based on the present findings, an appropriate awareness campaign that addresses the various issues associated with stigmatization should be designed. To strengthen and build resilient healthcare systems, health leaders and policymakers should consider and address the relevant sociocultural factors. In this regard, the present findings offer insights into the stigmatization processes operating during the COVID‐19 pandemic in Bangladesh. Decision‐makers should consider these factors when they develop strategies to manage similar health emergencies in Bangladesh.

### 4.1 Limitations of the study

This study has a few limitations. It was not possible to conduct in‐person interviews in all cases because of the pandemic. In addition, potential participants declined the invitation to participate in this study because of time limitations, a heavy workload, and unwillingness to be interviewed. Seven potential participants refused to participate in the interview due to COVID‐19‐related fears. Hospital staff members closely observed stigmatic behaviors. Therefore, they should be included in future studies to examine the responses of stigmatized individuals in greater depth.

## 5 CONCLUSION

Aiming to explore the transmission dynamics of the COVID‐19 through the stigmatization process among heterogeneous groups of people, this study concluded that diverse sociocultural factors influence the stigmatization. Study findings also revealed that stigmatization accelerates transmission. Healthcare decision‐makers and policymakers are trying to devise better strategies to manage this COVID‐19 pandemic that has already been identified as a global public health crisis. But without considering the sociocultural tradition, reality, and practices of the mass people, it is a complex issue to control and manage such a health burden. People understand and respond according to their interpretations, which develop from their contextual reality. It is evolved from this study that various sociocultural factors are the basis of contextual reality that influence stigmatization, ultimately contributing to increasing the transmission flow. A transdisciplinary approach is needed to handle this complex situation effectively. Transmission prevention and control are significant issues in such emerging or reemerging diseases where effective treatment is uncertain. People's perception, interpretation, and behaviors are vital here. Thus, issues related to the stigmatization process are very significant in preventing and controlling such health crises. The present findings may offer helpful insights to the relevant personnel and policymakers and help them in reexamining issues related to COVID‐19‐related stigmatization and reduce transmission flow. Further studies on an in‐depth exploration of such stigmatization processes and related issues will enrich our understanding of how such unexpected outbreaks can be effectively managed and controlled.

## FUNDING

Authors received no funding for this research.

## CONFLICTS OF INTEREST

The author declare that there is no conflict of interest.

## Data Availability

Interview guidelines are available in the supplementary files. Primary data cannot be opened publicly because of ethical limitations. Interested individuals may contact Mr. Jitu Mia (jitusust@gmail.com), Administrative Officer, Department of Anthropology, Shahjalal University of Science and Technology, for queries.
